# Biannual and Quarterly Comparison Analysis of Agglutinating Antibody Kinetics on a Subcohort of Individuals Exposed to *Leptospira interrogans* in Salvador, Brazil

**DOI:** 10.3389/fmed.2022.862378

**Published:** 2022-04-14

**Authors:** Jaqueline S. Cruz, Nivison Nery, Gielson A. Sacramento, Renato Victoriano, Albino L. S. Montenegro, Juliet O. Santana, Federico Costa, Albert I. Ko, Mitermayer G. Reis, Elsio A. Wunder

**Affiliations:** ^1^Laboratório de Patologia e Biologia Molecular, Instituto Gonçalo Moniz, Fundação Oswaldo Cruz, Ministério da Saúde, Salvador, Brazil; ^2^Instituto de Saúde Coletiva, Universidade Federal da Bahia, Salvador, Brazil; ^3^Departamento de Geografia, Instituto de Geociências, Universidade Federal da Bahia, Salvador, Brazil; ^4^Department of Epidemiology of Microbial Diseases, Yale School of Public Health, New Haven, CT, United States; ^5^Departamento de Medicina e Patologia Legal, Faculdade de Medicina da Bahia, Universidade Federal da Bahia, Salvador, Brazil

**Keywords:** *Leptospira*, leptospirosis, human, serosurvey, MAT, antibody kinetics

## Abstract

**Introduction:**

Leptospirosis is a zoonosis with a worldwide spread that leads to clinical manifestations ranging from asymptomatic infection to a life-threatening disease. The immune response is predominantly humoral mediated limited to the infecting serovar. Individuals living in an area endemic for leptospirosis are often exposed to an environment contaminated with leptospires and there is a paucity of information on naturally acquired immunity. In the present study, we evaluated the kinetics of agglutinating antibodies in individuals from an endemic area for leptospirosis in Salvador, Brazil comparing two different intersample collection times.

**Methods:**

Between 2017–2018, we carried out a biannual prospective cohort with 2,086 individuals living in an endemic area for leptospirosis in Salvador, Brazil. To compare agglutinating antibody kinetics using microscopic agglutination test (MAT) with different collection times, a subcohort of 72 individuals with quarterly follow-up was carried out in parallel.

**Results:**

The results revealed that using a shorter time for intersample collection led to the detection of a higher number of infections and reinfection events. Furthermore, we observed a higher rate of titer decay indicating partial and short protection. However, there was no indication of major changes in risk factors for the disease.

**Conclusions:**

We evaluated antibody kinetics among residents of an endemic area for leptospirosis comparing two sample collection times. The constant exposure to the contaminated environment increases the risk for leptospirosis infection with reinfection events being more common than expected. This indicates that the burden of leptospirosis might be underestimated by serological surveys, and further studies are necessary to better characterize the humoral response after infection.

## Introduction

Leptospirosis is a zoonosis of worldwide distribution and an important reemerging disease caused by pathogenic spirochetes of the genus *Leptospira* (1). The disease is endemic in a diverse range of epidemiological settings given the high number of animal reservoirs that can harbor the bacteria in their kidneys and excrete in their urine (2, 3). The transmission in humans occurs mainly through contact with environmental sources contaminated with the urine of infected animals. Rodents are the main source of human infection and responsible for the maintenance of the bacteria in the urban environment (4, 5). The clinical manifestations of the disease vary from asymptomatic or mild to severe disease such as Weil syndrome and pulmonary hemorrhage syndrome, associated with a lethality rate of 10 and 50%, respectively (3, 6–8).

The World Health Organization (WHO) estimates the occurrence of more than one million human cases of leptospirosis worldwide, with more than 50,000 deaths each year, most of which occurs in developing countries and tropical and subtropical climate regions (9, 10). In Brazil, 13,000 severe cases are reported per year with a lethality rate of 10.8% (11). The occurrence of urban epidemics, in Brazil and similar regions, is associated with environmental, occupational, and recreational risk factors and the large population of reservoirs (4, 12). More than 300 serovars can cause disease in humans and animals (13), but serovars from *L. interrogans* species are the most pathogenic and common throughout the world (14). In Brazil, serovar Copenhageni is the leading cause of epidemics in urban environments representing more than 90% of infections in Salvador with *Rattus norvegicus* (rat or sewer rat) as the main carrier (11, 15).

During the course of leptospirosis infection, the immune response is predominantly humoral mediated by the production of circulating antibodies directed against the lipopolysaccharide (LPS) and limited to the infecting serovar (16). Individuals living in regions where leptospirosis is endemic, are frequently being exposed to *Leptospira* and there are reports of the presence of anti-*Leptospira* antibodies in patients recovering from severe disease and in individuals with no previous history of the disease, most likely resulting from asymptomatic infection (17). However, there is little information on whether these individuals develop a naturally acquired protective immunity against infection or severe disease (17). In the present study we evaluated the kinetics of the humoral response after leptospirosis infection on individuals living in an urban slum area to obtain relevant information that may contribute to the better understanding of the naturally acquired immunity against leptospirosis reinfection.

## Materials and Methods

### Ethics Statement

This project was approved by the Research Ethics Committee of the Instituto Gonçalo Moniz, Fundação Oswaldo Cruz (FIOCRUZ), the National Research Ethics Council (CONEP) through the Certificate of Presentation for Ethical Appreciation (CAAE) #45217415.4.0000.0040 and the Yale University Institutional Review Board #1006006956. All participants received guidance on the objectives, procedures, and risks associated with participation during informed consent. Minors gave verbal consent and we obtained written consent by their parents or legal guardian. Collegiality of participation was assured. Laboratory results were made available to the cohort participants.

### Study Site and Population

**Figure 1:**
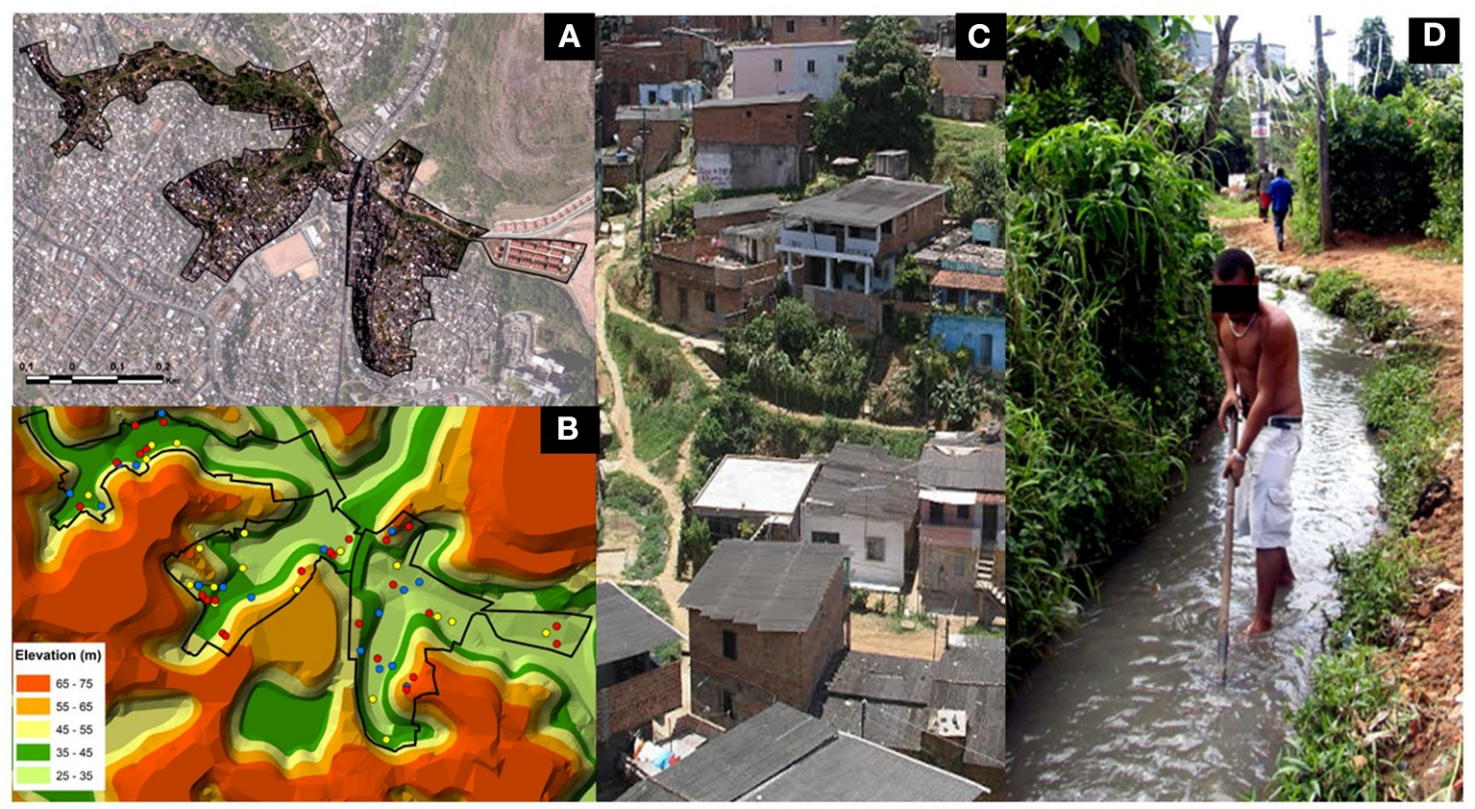
Slum community site in the city of Salvador, Brazil. **(A)** Aerial photograph showing the study site boundary in the community of Pau da Lima, Brazil. **(B)** Topographic map highlighting the households participating in the subcohort study. In blue, households with uninfected individuals; in yellow, households with infected individuals and in red, households with reinfected individuals. Photographs showing social characteristics **(C)** and environmental risk factors **(D)** of the community.

A prospective cohort study was carried out in the community of Pau da Lima (13° 32′53.47 “S: 38° 43′51.10” W), an urban slum community of Salvador, Bahia, Brazil (Figure 1). This community was described as an area of 0.24 km^2^ made up of three valleys, with open sewage close to the residences, places of garbage accumulation, and risk of flooding mainly in the bottom areas of the valleys (Figure 1). Due to the irregular occupation of the land and the precarious urban infrastructure, the community of Pau da Lima presents similar conditions to other vulnerable communities in Brazil and tropical regions of the world (9, 18). Previous studies have shown that residents of this community are in contact with the contaminated environment throughout the year leading to a high risk of leptospirosis infection facilitated by rat infestation, contact with mud promoted by topographic factors such as home elevation and inadequate drainage (9, 19). The crude rate of *Leptospira* infection in this community was 37.8 per 1,000 person-years with a 2.3-fold higher rate of secondary infection when compared to the rate of primary infection (19).

Between September 2017 to December 2018, residents of Pau da Lima with ≥5 years and who slept at least three nights a week at home were enrolled in the biannual analysis study. During all visits (every 6 months), blood samples were collected for evaluation of anti-leptospire antibodies using the microscopic agglutination test (MAT). Previously validated epidemiological, exposure and sociodemographic questionnaires were applied annually for field data collection. At the moment of enrollment for the biannual study, individuals with a previous history of leptospirosis infection determined by MAT were enrolled for our subcohort, together with controls that had no history of infection. For this subcohort, a quarterly analysis follow-up was performed (every 3 months) for blood collection, with a total of five home visits to assess antibody kinetics. For the entire study, we considered as exclusion criteria the following: participant not found at the time of the team visit, refusal to participate in the study and inability to locate the participants after three attempts.

### Serologic Evaluation for *Leptospira* Infection

The blood samples collected were sent to the Laboratory of Pathology and Molecular Biology at Instituto Gonçalo Moniz, Fiocruz, BA. Sera was obtained through centrifugation, and it was processed for the presence of agglutinating antibodies against *Leptospira* using the microscopic agglutination test (MAT). The MAT was performed with *L. interrogans* serovar Copenhageni strain Fiocruz L1-130, the most prevalent serovar in the region (9, 15, 18, 19). The screening was performed using 1:50 and 1:100 dilutions of serum. A positive sample was determined when agglutination was observed on more than 50% of leptospires compared to the control with no sera. If the sera were positive at the dilution of 1:100, the sample was titrated to determine the highest agglutination titer. The presence of anti-*Leptospira* agglutinating antibodies was used as a marker for previous infection. A case of *Leptospira* infection was defined as individuals with antibody titers ≥1:50 at any time-point, and/or with a seroconversion between consecutive time-points, defined as the absence of agglutination reaction in the first sample and the presence of agglutination with ≥1:50 in the following sample, and/or four-fold rise in titer between consecutive time-points. Reinfection was defined as participants who had two or more infections documented based on the MAT results during the follow-up period.

### Statistical Analyses

Statistical analyses were performed using the RStudio package, version 1.2.5033. Descriptive analysis was performed to obtain absolute frequencies or means and medians for categorical variables and univariate analysis through Welch's two-sample *t*-test and Pearson's chi-square test, with 95% CI. Statistical significance was considered when the probability value *p* ≤ 0.05. GraphPad Prism version 7 for Windows was used to evaluate the kappa coefficient of agreement between collection times and the kinetic data calculated in log10 and plotted against collection periods. A logistic regression model was used to determine the adjusted odds ratio (OR) (95% confidence intervals) to assess whether MAT titers protect against reinfection.

## Results

### Enrollment and Follow-Up of Study Participants

**Figure 2:**
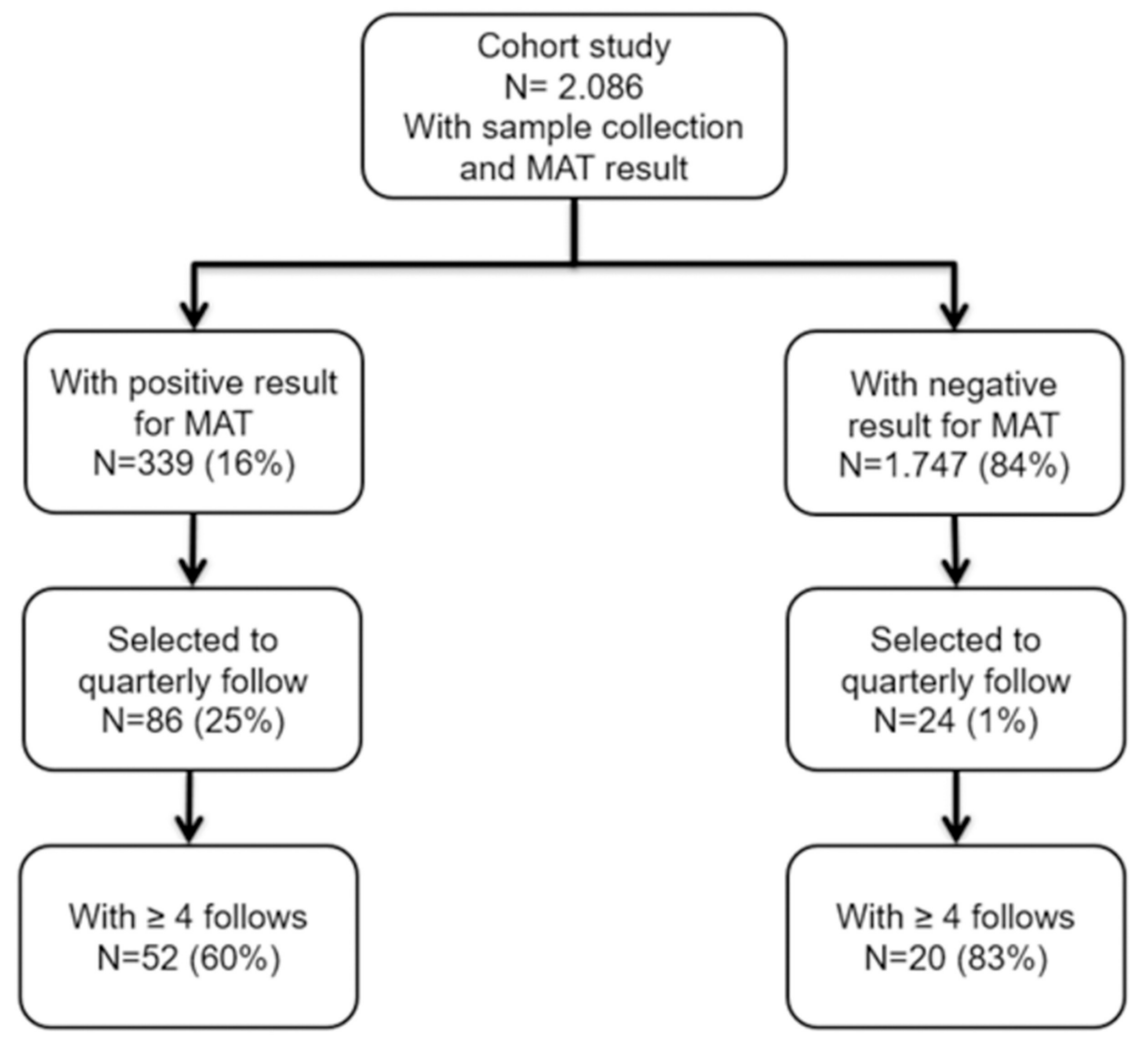
Flowchart of the study participants enrollment.

During a cohort study for leptospirosis conducted from 2017–2018, a total of 2,086 individuals were enrolled for a biannual collection of blood for MAT assay and analysis of epidemiological data. Among them, 339 (16%) were serologically confirmed for leptospirosis infection. We enrolled 110 participants for a subcohort performing a quarterly analysis follow-up. Among those, 52 confirmed cases and 20 negative cases that had ≥4 blood samples at the end of the study were analyzed (Figure 2).

### Characteristic of the Participants

The demographic, socioeconomic and exposure information of all the cohort participants and the subcohort are shown in Table 1. Most of the participants were young adults with a median age between 27–28 years old, with 57.8% of women for the cohort and 50% for the subcohort, respectively. Regarding ethnicity, there was a predominance of brown race (46.5% for the cohort and 45.8% for the subcohort) and <9 years of education was reported in all groups (78.2 and 77.8%, respectively). Informal employment was described by 36.6% of the cohort and 50% of the subcohort participants. Construction work (8.6% for the cohort and 6.9% for the subcohort) and work related to garbage removal (3.5 and 9.7%, respectively) were most described activities in the groups. The median per capita household income was similar among groups, with US$ 4.7 daily for the cohort and US$ 4.3 daily for the subcohort. Among the exposure variables, in all groups open sewage was reported <10 m from the house (70.2 and 69.4%), contact with sewage (37.2 and 45.8%), contact with floodwater near home (39.4 and 55.6%), contact with mud near home (45.5 and 62.5%) and 14.9% of the total cohort reported cleaning sewage followed by 13.9% of the subcohort group.

**Table 1:** Sociodemographic and exposure characteristics of the participants enrolled in the study.

**Characteristic**	**Cohort total** **(***N*** = 2,086)^***a***^**	**Subcohort** **(***N*** = 72)^***a***^**
Median Age (years)	27 (17)	28 (16)
Age (years)		
05–14	631 (30.2%)	21 (29.2%)
15–24	404 (19.4%)	9 (12.5%)
25–34	399 (19.1%)	18 (25.0%)
35–44	292 (14.0%)	12 (16.7%)
> 44	360 (17.3%)	12 (16.7%)
Sex		
Female	1,206 (57.8%)	36 (50.0%)
Male	880 (42.2%)	36 (50.0%)
Ethnicity		
Black	944 (45.3%)	29 (40.3%)
Brown	969 (46.5%)	33 (45.8%)
White	154 (7.4%)	10 (13.9%)
Others	19 (0.9%)	0 (0.0%)
Education		
Up to 9th year	1,632 (78.2%)	56 (77.8%)
More than 9th year	454 (21.8%)	16 (22.2%)
Married or stable union	730 (35.0%)	22 (30.6%)
Informal employment	763 (36.6%)	36 (50.0%)
Per capita household income (US$/day)	4.7 (4.8)	4.3 (3.7)
Cleaned sewage	311 (14.9%)	10 (13.9%)
Open sewage at <10 m from home	1,464 (70.2%)	50 (69.4%)
Accumulated trash within <10 m of home	781 (37.4%)	24 (33.3%)
Sewage contact	777 (37.2%)	33 (45.8%)
Floodwater near home	822 (39.4%)	40 (55.6%)
Mud near home	949 (45.5%)	45 (62.5%)
Work in construction	179 (8.6%)	5 (6.9%)
Work related to hawker	47 (2.3%)	3 (4.2%)
Work related to garbage removal	74 (3.5%)	7 (9.7%)
Work involves contact with mud	60 (2.9%)	3 (4.2%)
Work involves contact with flood water	50 (2.4%)	3 (4.2%)
Work involves sewage contact	44 (2.1%)	3 (4.2%)
Fever	559 (26.8%)	16 (22.2%)

a *Median (IQR); n (%)*.

**Figure 3:**
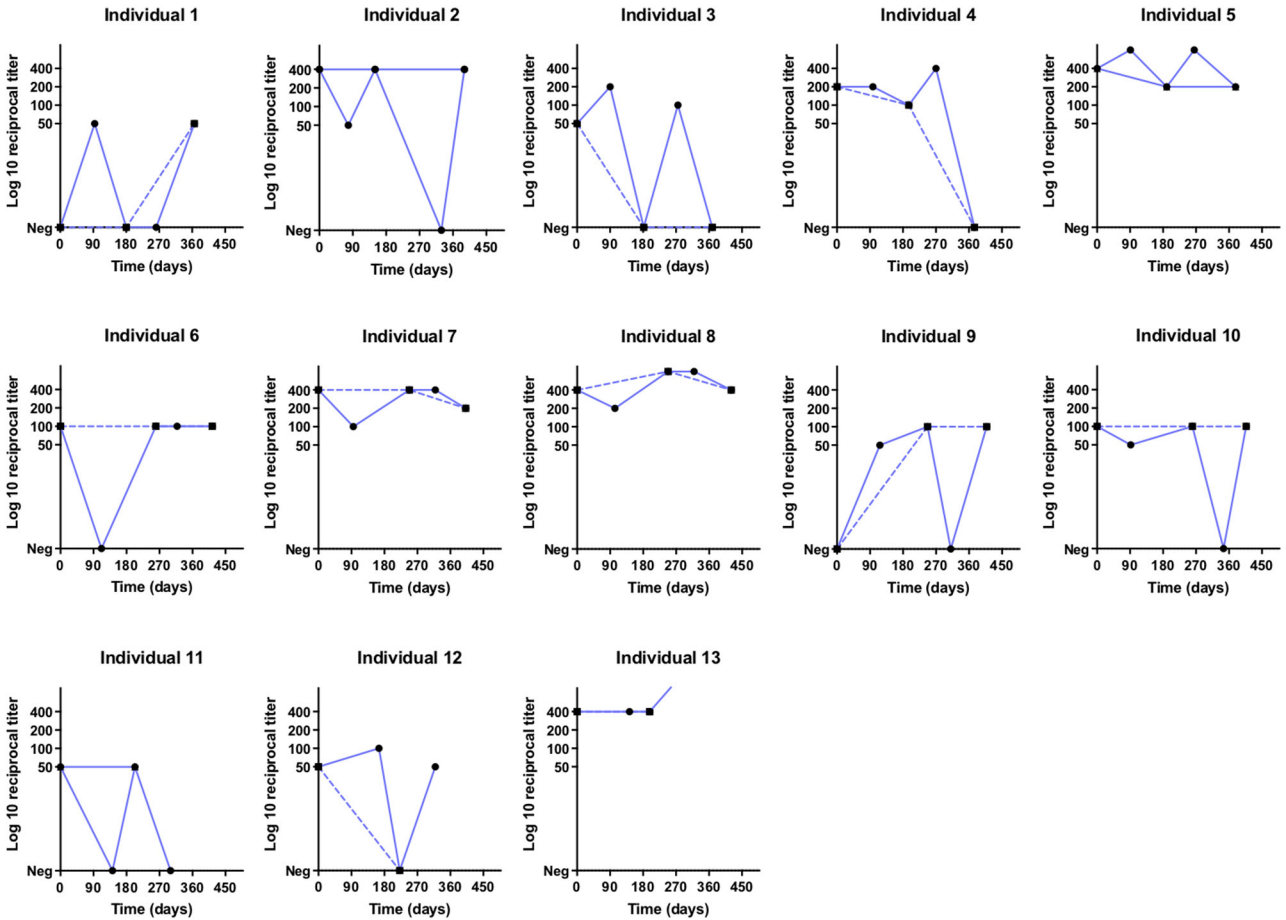
Titration curves in log10 of individuals classified as reinfection in the quarterly analysis (∙) and infection in the biannual analysis (▪) at different times (days) of collection.

The kappa statistics for leptospirosis case classification at the different collection times was 0.48 (95% CI: 0.32–0.63), achieving moderate agreement (Table 2). In this evaluation we identified differences in leptospirosis case classifications when comparing biannual analysis and quarterly analysis collections, mainly between the infected vs. reinfected and no-infected vs. infected groups. When performing a biannual analysis, we identified 25 (34.7%) infections, 12 (16.6%) reinfections and 35 (48.6%) negative individuals, while in the quarterly analysis, we identified 22 (30.5%) infections, and 25 (34.7%) reinfections and non-infections, each. There are 13 (18%) individuals that would be classified as reinfection rather than infection when performing a quarterly analysis (Table 2, Figure 3). Furthermore, the quarterly analysis identified an extra 8 (11%) individuals as infection and 2 (2.8%) individuals as reinfection rather than no-infection determined by the biannual analysis (Table 2, Supplementary Figure S1). In contrast, there were only 2 individuals classified as reinfection by the biannual analysis that would be considered as infection by the quarterly evaluation (Table 2, Supplementary Figure S1). The multivariate analysis did not find an association between MAT titers and reinfection (Table 3). Taken together, those results indicate that the decay of agglutinating antibodies is shorter than expected in individuals exposed to leptospires without severe symptoms, and the time between assessment of those antibodies can influence the number of infections and reinfections. Further, our data suggests that agglutinating antibodies might not be the ideal correlates for naturally acquired immunity against reinfection.

**Table 2:** Concordance between the biannual and quarterly follow-up collections.

**Quarterly follow-up**		**Biannual follow-up**			
		**Infection**	**Reinfection**	**No infection**	**Total**
	Infection	12	2	8	22
	Reinfection	13	10	2	25
	No Infection	0	0	25	25
	Total	25	12	35	72

**Table 3:** Multivariate analysis to evaluate MAT titers as immune markers for reinfection on biannual and quarterly follow-up.

	**Biannual analysis (*****N*** **= 7)**			**Quarterly analysis (*****N*** **= 7)**		
**Characteristic**	**OR^***a***^**	**95% CI^***b***^**	***p*** **-value**	**OR^***a***^**	**95% CI^***b***^**	***p*** **-value**
(Intercept)	0.96	0.33 – 2.80	0.941	0.82	0.29 – 2.26	0.701
Sewage contact						
No	—	—		—	—	
Yes	1.08	0.33 – 3.54	0.894	1.26	0.37 – 4.43	0.713
Titers of MAT						
≤ 100	—	—		—	—	
≥200	2.33	0.49 – 13.35	0.303	4.26	0.52 – 90.63	0.226
400	0.8	0.16 – 3.94	0.786	1.69	0.45 – 6.72	0.438
≥800	1.01	0.19 – 5.44	0.991	0.34	0.02 – 3.21	0.386

a *OR = Odds Ratio*.

b *CI = Confidence Interval*.

### Agreement Analysis

The distribution of MAT titers of samples positive for the Fiocruz L1-130 strain and their frequencies are listed in Supplementary Table S1. Of the total, 47 (65%) had anti-leptospire agglutinins in the quarterly analysis collection while 37 (51%) in the biannual analysis collection. Most participants in the group with quarterly analysis collection had low titers, with 62% ranging from 1:50 (28%) to 1:200 (21%). The highest agglutination titers in the biannual analysis collection period were observed at the 1:400 dilution (41%). Those results indicate that most of the exposures lead to low agglutinating titers.

### Biannual vs. Quarterly Follow-Up Analysis

We then evaluated the characteristics of the subcohort participants stratified by infection and non-infection based on different collection times (biannual vs. quarterly analysis) (Table 4). The variables age (*p* < 0.001 and *p* = 0.005) and cleaning sewage (*p* = 0.003 and *p* = 0.033) were associated with a higher chance of infection for both groups with different collection times. Furthermore, informal employment (*p* = 0.013) for the quarterly analysis collection group and being married or having a stable union (*p* = 0.032) for the biannual were also associated with a higher chance of infection when compared to the non-infection group (Table 4). A more detailed analysis of the participants per collection time stratifying the infection group by single infection and reinfection event showed that cleaning sewage was the only significant risk for infection (*p* = 0.04) on the quarterly analysis group (Supplementary Table S2). For reinfection, age (*p* = 0.03) and informal employment (*p* = 0.01) were both associated with a higher risk (Supplementary Table S2). A similar analysis using the biannual analysis data showed that variables age and cleaning sewage were both significant risks for infection and reinfection (Supplementary Table S3). Interestingly, having a married or stable union (*p* = 0.047) showed as a risk for infection on the biannual analysis (Supplementary Table S3). There was no statistically significant difference when comparing the infection and reinfection groups in both analyses with different collection times. Those results showed that despite the differences of infections and reinfections rates among the two different analyses, the risk for overall infection is similar in both groups, indicating that the time between serological evaluation might not have a major impact on the outcome of risk analysis.

**Table 4:** Comparison of the sociodemographic and exposure characteristics of the individuals in the subcohort based on the biannual analysis and quarterly analysis follow-up.

**Characteristic**	**Biannual (*****N*** **= 72)**			**Quarterly (*****N*** **= 72)**		
	**No infection** **(***N*** = 35)^***a***^**	**Infection** **(***N*** = 37)^***a***^**	***p*** **-value^***b***^**	**No infection** **(***N*** = 25)^***a***^**	**Infection** **(***N*** = 47)^***a***^**	***p*** **-value^***b***^**
Age (years)			**<0.001**			**0, 005**
05–14	16 (45.7%)	5 (13.5%)		13 (52)	8 (17)	
15–24	4 (11.4%)	5 (13.5%)		2 (8.0)	7 (15)	
25–34	4 (11.4%)	14 (37.8%)		3 (12)	15 (32)	
35–44	2 (5.7%)	10 (27.0%)		1 (4.0)	11 (23)	
> 44	9 (25.7%)	3 (8.1%)		6 (24)	6 (13)	
Sex			>0.99			>0.99
Female	17 (48.6%)	19 (51.4%)		13 (52)	23 (49)	
Male	18 (51.4%)	18 (48.6%)		12 (48)	24 (51)	
Ethnicity			0, 34			0, 17
Black	13 (37.1%)	16 (43.2%)		8 (32)	21 (45)	
Brown	15 (42.9%)	18 (48.6%)		11 (44)	22 (47)	
White	7 (20.0%)	3 (8.1%)		6 (24)	4 (8.5)	
Others	0 (0.0%)	0 (0.0%)		0 (0)	0 (0)	
Education			0, 87			0, 53
Up to 9th year	28 (80.0%)	28 (75.7%)		21 (84)	35 (74)	
More than 9th year	7 (20.0%)	9 (24.3%)		4 (16)	12 (26)	
Married or stable union	6 (17.1%)	16 (43.2%)	**0, 032**	4 (16)	18 (38)	0, 092
Informal employment	14 (40.0%)	22 (59.5%)	0, 16	7 (28)	29 (62)	**0, 013**
Per capita household income (US$/day)	4.2 (4.1)	4.5 (3.3)	0, 67	3.9 (4.4)	4.6 (3.3)	0, 52
Cleaned sewage	0 (0.0%)	10 (27.0%)	**0, 003**	0 (0)	10 (21)	**0, 033**
Open sewage at <10 m from home	21 (60.0%)	29 (78.4%)	0, 15	15 (60)	35 (74)	0, 32
Accumulated trash within <10 m of home	11 (31.4%)	13 (35.1%)	0, 93	7 (28)	17 (36)	0, 66
Sewage contact	15 (42.9%)	18 (48.6%)	0, 8	12 (48)	21 (45)	0, 98
Floodwater near home	21 (60.0%)	19 (51.4%)	0, 62	15 (60)	25 (53)	0, 76
Mud near home	20 (57.1%)	25 (67.6%)	0, 5	15 (60)	30 (64)	0, 95
Work in construction	2 (5.7%)	3 (8.1%)	>0.99	1 (4.0)	4 (8.5)	0, 82
Work related to hawker	1 (2.9%)	2 (5.4%)	>0.99	1 (4.0)	2 (4.3)	>0.99
Work related to garbage removal	1 (2.9%)	6 (16.2%)	0, 13	1 (4.0)	6 (13)	0, 44
Work involves contact with mud	1 (2.9%)	2 (5.4%)	>0.99	1 (4.0)	2 (4.3)	>0.99
Work involves contact with flood water	1 (2.9%)	2 (5.4%)	>0.99	1 (4.0)	2 (4.3)	>0.99
Work involves sewage contact	1 (2.9%)	2 (5.4%)	>0.99	1 (4.0)	2 (4.3)	>0.99
Fever	5 (14.3%)	11 (29.7%)	0, 2	4 (16)	12 (26)	0, 53

a *Mean (SD) or Frequency (%)*.

b *Welch Two Sample t-test; Pearson's Chi-squared test*.

*The values in bold are the variables with statistical significance (≤ 0.05)*.

## Discussion

The immunity against leptospirosis is based on a short-term humoral response for humans and animals (14). However, the few existing studies that report the kinetics of antibodies were performed in clinical patients associated with disease severity (20, 21) or in experimental animal models (14, 22). Evaluating antibody kinetics in individuals with natural *Leptospira* infection will help to better understand the duration of the immune response after infection, the course of infection, and the dynamics of protective immunity. In this study, we had the opportunity to evaluate the kinetics of the antibody response and the factors associated with exposure to leptospirosis in naturally infected individuals with asymptomatic infection comparing a biannual and a quarterly serological analysis. Among the 72 individuals who participated in the subcohort with ≥4 quarterly collections, we found that 65% had circulating anti-*Leptospira* antibodies. The variables age (*p* < 0.001 and *p* = 0.005) and cleaning sewage (*p* = 0.003 and *p* = 0.033) were associated with a higher chance of infection in both analyses.

The time of blood collection between samples can affect the number of infection and reinfection of leptospirosis. A recent study applied a titer decay rate on the serological data of the same population in Salvador, Brazil and identified a higher number of mean infection rate on the biannual analysis and even higher when applying the decay to annual analysis (23). In agreement with this report, our quarterly serological analysis identified a higher number of leptospirosis infections and reinfections events in our population when compared with a biannual analysis, suggesting that exposures to the leptospirosis pathogen in this urban slum setting are frequent if not ubiquitous. Given the constant high risk for exposure to the pathogen observed in this community either by the environment (9, 18) or by the high mobility of its inhabitants (24) and the bias that a reexposure and potential boost of titers can do to titers decay (23), it might be impossible to calculate an accurate titer decay. This limitation can affect the correct incidence rate, data comparison among different longitudinal cohort studies and potentially risk assessment for exposure. Further considerations should be made on reducing the time of serological evaluation or applying decay rates estimations to take into account the differences observed here on titer decay.

Our results indicate that the humoral response detected by MAT is relatively short and provides partial protection against reinfection. Previous studies have reported that individuals with leptospirosis were protected against reinfection by the same *Leptospira* serovar or by related serovars for a short period (25, 26). However, a recent study from French Polynesia showed that individuals with a first infection might not be protected against subsequent reinfection (27). In our study, when performing a quarterly analysis, we observed that agglutinating antibodies have a short life span with titers up to 1:200 disappearing after 90 days. Also, our analysis showed that agglutinating antibodies don't seem to affect the risk for subsequent infection. These results are in agreement with previous data that showed that constant exposure and pre-existing anti-leptospire antibodies did not provide complete immunity (19). Of note, most of the titers observed in our quarterly analysis were low, with 62% ranging between 1:50 and 1:200. A recent study of an attenuated vaccine has shown that antibodies against proteins rather than agglutinating antibodies are correlated to protection (28). It is possible that individuals in this community, which are often being exposed to an environment contaminated with leptospires, have built an immune response similar to a live vaccine that reduces symptoms in case of reinfection and potentially providing cross-protection between unrelated *Leptospira* serovars (5, 29, 30). Further studies to better characterize the immune response after infection, focusing on B and T cell responses and memory, would provide valuable information about potential markers to protect against reinfection.

The time of sample collection and the higher infection rates don't seem to affect the major risk factors for leptospirosis infection. Despite the assumption that a more suitable analysis leading to higher infection rates could potentially affect the observed risk factors for the disease (23) our results indicate that regardless of the period of analysis the potential risks for infection are similar. Transmission dynamics and risk factors for *Leptospira* infection and reinfection are associated with environmental, demographic, and individual exposures. Our results show that risk factors for infection in this community corroborate previous studies (9, 18, 19). The chance of acquiring anti-leptospire antibodies was more frequent in young adults with <9 years of schooling, regardless of the time of collection. Although gender was not identified as a risk factor for acquiring infection in our study, several others consistently report that men in working age groups are at higher risk (9, 10, 15, 31, 32). In our study, being married or having a stable relationship was associated with a risk of infection. We also found that in this group 69% (11/16) of individuals with infection were women against 25% (4/16) of men. A study in Cali, Colombia, showed that the female gender was directly associated with the risk of *Leptospira* infection and that domestic factors may play an important role in transmission, particularly in urban slums (33, 34). Another possibility to explain this finding is related to factors such as age and exposure time. We observed that the general mean age in the group of married individuals with infection was 33 years, while for single individuals with infection it was 27 years. Our findings may be explained by the fact that being older resulted in longer exposure time and a greater risk of infection. Recently, a study on the same area showed that increasing age was associated with an increased risk of *Leptospira* infection, and that infections in this area can occur year-round (35). Infection is also often associated with occupational activity such as working in civil construction, working with garbage removal, and informal employment (9), the latter was also identified in our study. We also identified individual exposures related to the home environment such as contact with mud, standing water in the vicinity of the home, and especially sewage cleaning, which was associated with an increased risk of infection in both analysis and has been reported as a risk factor for *Leptospira* infection (9, 19).

This study has some limitations that should be considered. The sample size of the quarterly analysis was not ideal for some of our analysis. Longitudinal cohort analysis are logistically and financially troublesome, which is reflected on the few studies conducting such experiments on leptospirosis and the choice to make biannual or annual measurements (9, 18, 19). For that reason, we decided to select a sample of participants who had confirmed infection at the time the biannual survey was carried out. In addition, 35% of subjects in the subcohort did not complete quarterly follow-up, primarily due to moving out of the study area, which is a common issue in longitudinal studies. Despite those limitations, we were able to identify significant differences in our analysis that agreed with previous studies, indicating the validity of our results. The MAT is the gold standard test recognized by the WHO, but it is a laborious test, subjective and requires experience from the reader. Further, the MAT does not differentiate past from current infection. Those limitations from the MAT are a common feature for several serological assays and always present on leptospirosis studies (30, 36, 37). To minimize impacts on MAT results in our study, only a well-trained and experienced technician was responsible for all readings. Our group has been working in Salvador, Brazil and in the community of Pau da Lima, where this study was conducted, for over 20 years. Since then, we have reduced our panel of MAT strains given the extensive knowledge of circulating strains and reservoirs (9, 18, 19). Our previous studies have shown that over 90% of severe cases of leptospirosis (15) and 90–98% of infections in the community are related to *L. interrogans* serovar Copenhageni. Furthermore, 80% of rats captured in the community (9, 18, 19, 38) were culture positive for leptospirosis, and the serovar Copenhageni was the only one isolated (39). The focus of our study was to understand the role of agglutinating antibodies on the naturally acquired immunity against reinfection, and to have statistical power our analysis were based on the most prevalent serovar in our study site, *L. interrogans* serovar Copenhageni. For those reasons we didn't evaluate agglutinating antibodies for other serovars, including *L. biflexa* serovar Patoc, commonly used as a control. Titers of 1:25 or 1:50, as well as higher titers, were directed against this serovar in our precious studies (9, 18, 19), indicating that this cutoff was a specific and more sensitive criteria for identifying prior infections in a region where a single serovar agent is circulating. Our study site has geographical and social-demographic features that are very similar to other regions of the world where leptospirosis is a problem. Furthermore, the *L. interrogans* is the most common species related to human cases of leptospirosis around the globe (14). Although our results can be generalized to the context of urban leptospirosis worldwide, considerations should be made given recent reports in a mice model that different strains can lead to different levels of immune responses (14).

In summary, we reported antibody kinetics in individuals from an endemic area for leptospirosis showing that frequent exposure to the contaminated environment is an important factor on the infection and reinfection rates of the disease, which are directly affected by the time of intersample collection. Our study also suggested a rapid decay of the humoral response related to agglutinating antibodies and a short-lived naturally acquired immunity against reinfections. Furthermore, our results indicated that serological surveys may be underestimating the burden of *Leptospira* infection and potentially the risk for disease. Further studies are needed to evaluate memory B cells and to assess the humoral response of individuals with previous leptospirosis infections, that could help better to understand the naturally acquired immunity of this important neglected disease and close the knowledge gap on correlates of immunity that can be used to improve prevention.

## Data Availability Statement

The original contributions presented in the study are included in the article/supplementary materials, further inquiries can be directed to the corresponding author.

## Ethics Statement

The studies involving human participants were reviewed and approved by Yale University Institutional Review Board Research Ethics Committee of the Instituto Gonçalo Moniz Certificate of Presentation for Ethical Appreciation (CAAE). Written informed consent to participate in this study was provided by the participants or their legal guardian/next of kin.

## Author Contributions

JC: conception, methodology, and writing of the original draft. NN: data curation, data analysis, and review. GS, RV, AM, and JS: investigation and review. FC, AK, and MR: funding acquisition, investigation, methodology, and review. EW: funding acquisition, investigation, methodology, proofreading, supervision, writing-proofreading, and editing in English. All authors contributed to the revision and editing of the manuscript, and approved the submitted version.

## Funding

This research was funded by grants from the National Institutes of Health (R01AI052473, U01AI088752, R01TW009504, R25TW009338, and R01AI121207), Well-come Trust (218987/Z/19/Z), Coordination for the Improvement of Higher Education (CAPES), from Brazil, and National Council for Scientific and Technological Development (CNPq) (307319/2016-4). The funders had no role in study design, data collection and analysis, decision to publish, or preparation of the manuscript.

## Conflict of Interest

The authors declare that the research was conducted in the absence of any commercial or financial relationships that could be construed as a potential conflict of interest.

## Publisher's Note

All claims expressed in this article are solely those of the authors and do not necessarily represent those of their affiliated organizations, or those of the publisher, the editors and the reviewers. Any product that may be evaluated in this article, or claim that may be made by its manufacturer, is not guaranteed or endorsed by the publisher.

## Supplementary Material

Supplementary Figure S1 Titration curves in log 10 of individuals classified in the quarterly analysis (▪) as infection (Individual 14 to individual 21) and reinfection (Individuals 22 and 23) at different times (days) of collection, compared to the bi-annual analysis (∙).

Supplementary Table S1 Anti-leptospira antibody titers distributed by classification and collection period.

Supplementary Table S2 Characteristic of the subcohort group comparing the stratified analysis of infection based on the quarterly analysis follow-up.

Supplementary Table S3 Characteristic of the subcohort group comparing the stratified analysis of infection based on the biannual analysis follow-up.
