# Supplementary figures and images for: Biannual and Quarterly Comparison Analysis of Agglutinating Antibody Kinetics on a Subcohort of Individuals Exposed to Leptospira interrogans in Salvador, Brazil
Source: Front Med (Lausanne). 2022 Apr 14;9:862378. doi: 10.3389/fmed.2022.862378 (PMC9048256; doi:10.3389/fmed.2022.862378)

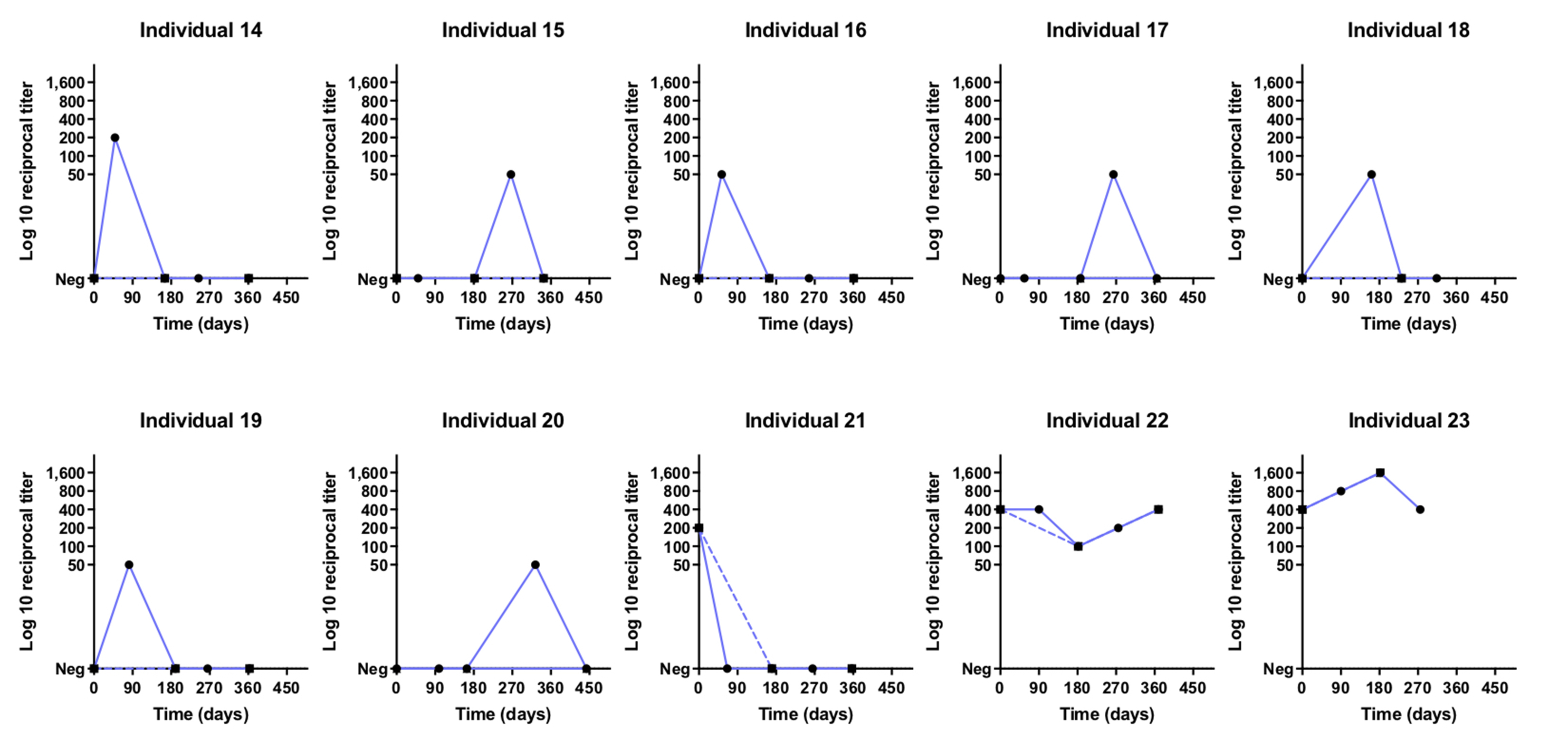

Supplement: Supplementary Figure S1 — Titration curves in log 10 of individuals classified in the quarterly analysis (▪) as infection (Individual 14 to individual 21) and reinfection (Individuals 22 and 23) at different times (days) of collection, compared to the bi-annual analysis (∙). [file Image_1.JPEG]
