# Supplementary material for: Biannual and Quarterly Comparison Analysis of Agglutinating Antibody Kinetics on a Subcohort of Individuals Exposed to Leptospira interrogans in Salvador, Brazil
Source: Front Med (Lausanne). 2022 Apr 14;9:862378. doi: 10.3389/fmed.2022.862378 (PMC9048256; doi:10.3389/fmed.2022.862378)
Supplement: Supplementary Table S1 — Anti-leptospira antibody titers distributed by classification and collection period. [file Table_1.DOCX]

Supplementary Table 1. Anti-leptospira antibody titers distributed by classification and collection period.

|  | **MAT Titer at the time of infection** | | | | | | |
| --- | --- | --- | --- | --- | --- | --- | --- |
|  | **n (%)** | | | | | | |
|  | **Total in Infections** | 1:50 | 1:100 | 1:200 | 1:400 | 1:800 | ≥ 1:1600 |
| Biannual follow-up | 37/72 (51) | 7 (19) | 6 (16) | 5 (14) | 15 (41) | 2 (5) | 2 (5) |
| Quarterly follow-up | 47/72 (65) | 13 (28) | 6 (13) | 10 (21) | 10 (21) | 6 (13) | 2 (4) |
