# Supplementary material for: Biannual and Quarterly Comparison Analysis of Agglutinating Antibody Kinetics on a Subcohort of Individuals Exposed to Leptospira interrogans in Salvador, Brazil
Source: Front Med (Lausanne). 2022 Apr 14;9:862378. doi: 10.3389/fmed.2022.862378 (PMC9048256; doi:10.3389/fmed.2022.862378)
Supplement: Supplementary Table S2 — Characteristic of the subcohort group comparing the stratified analysis of infection based on the quarterly analysis follow-up. [file Table_2.DOCX]

Supplementary Table 2. Characteristic of the subcohort group comparing the stratified analysis of infection based on the quarterly analysis follow-up.

| **Characteristic** | **Total (N = 72)^1^** | **No infection (N = 25)^1^** | **Infection (N = 22)^1^** | **Reinfection (N = 25)^1^** | **p-value^2^ infection vs no infection** | **p-value^2^ reinfection vs no infection** | **p-value^2^**  **infection vs reinfection** |
| --- | --- | --- | --- | --- | --- | --- | --- |
| **Mean Age (years)** | 28 (16) | 24 (19) | 26 (15) | 34 (13) | 0,63 | **0,034** | 0,058 |
| **Age (years)** |  |  |  |  | 0,091 | **0,002** | 0,18 |
| 05-14 | 21 (29) | 13 (52) | 6 (27) | 2 (8.0) |  |  |  |
| 15-24 | 9 (12) | 2 (8.0) | 5 (23) | 2 (8.0) |  |  |  |
| 25-34 | 18 (25) | 3 (12) | 5 (23) | 10 (40) |  |  |  |
| 35-44 | 12 (17) | 1 (4.0) | 4 (18) | 7 (28) |  |  |  |
| > 44 | 12 (17) | 6 (24) | 2 (9.1) | 4 (16) |  |  |  |
| **Sex** |  |  |  |  | 0,88 | >0.99 | 0,88 |
| Female | 36 (50) | 13 (52) | 10 (45) | 13 (52) |  |  |  |
| Male | 36 (50) | 12 (48) | 12 (55) | 12 (48) |  |  |  |
| **Ethnicity** |  |  |  |  | 0,67 | 0,092 | 0,37 |
| Black | 29 (40) | 8 (32) | 8 (36) | 13 (52) |  |  |  |
| Brown | 33 (46) | 11 (44) | 11 (50) | 11 (44) |  |  |  |
| White | 10 (14) | 6 (24) | 3 (14) | 1 (4.0) |  |  |  |
| Others | 0 (0) | 0 (0) | 0 (0) | 0 (0) |  |  |  |
| **Education** |  |  |  |  | 0,35 | >0.99 | 0,55 |
| Up to 9th year | 56 (78) | 21(84) | 15 (68) | 20 (80) |  |  |  |
| More than 9th year | 16 (22) | 4 (16) | 7 (32) | 5 (20) |  |  |  |
| **Married or stable union** | 22 (31) | 4 (16) | 8 (36) | 10 (40) | 0,21 | 0,12 | >0.99 |
| **Informal employment** | 36 (50) | 7 (28) | 12 (55) | 17 (68) | 0,12 | **0,011** | 0,52 |
| **Per capita household income (US$/day)** | 4.3 (3.7) | 3.9 (4.4) | 4.2 (3.0) | 4.9 (3.5) | 0,78 | 0,41 | 0,51 |
| **Cleaned sewage** | 10 (14) | 0(0) | 5 (23) | 5 (20) | **0,041** | 0,059 | >0.99 |
| **Open sewage at <10m from home** | 50 (69) | 15 (60) | 15 (68) | 20 (80) | 0,78 | 0,22 | 0,55 |
| **Accumulated trash within <10m of home** | 24 (33) | 7 (28) | 8 (36) | 9 (36) | 0,76 | 0,76 | >0.99 |
| **Sewage contact** | 33 (46) | 12 (48) | 9 (41) | 12 (48) | 0,85 | >0.99 | 0,85 |
| **Floodwater near home** | 40 (56) | 15 (60) | 10 (45) | 15 (60) | 0,48 | >0.99 | 0,48 |
| **Mud near home** | 45 (62) | 15 (60) | 14 (64) | 16 (64) | >0.99 | >0.99 | >0.99 |
| **Work in construction** | 5 (6.9) | 1 (4.0) | 2 (9.1) | 2 (8.0) | 0,91 | >0.99 | >0.99 |
| **Work related to hawker** | 3 (4.2) | 1 (4.0) | 2 (9.1) | 0 (0) | 0,91 | >0.99 | 0,41 |
| **Work related to garbage removal** | 7 (9.7) | 1 (4.0) | 2 (9.1) | 4 (16) | 0,91 | 0,35 | 0,79 |
| **Work involves contact with mud** | 3 (4.2) | 1 (4.0) | 1 (4.5) | 1 (4.0) | >0.99 | >0.99 | >0.99 |
| **Work involves contact with flood water** | 3 (4.2) | 1 (4.0) | 1 (4.5) | 1 (4.0) | >0.99 | >0.99 | >0.99 |
| **Work involves sewage contact** | 3 (4.2) | 1 (4.0) | 1 (4.5) | 1 (4.0) | >0.99 | >0.99 | >0.99 |
| **Fever** | 16 (22) | 4 (16) | 8 (36) | 4 (16) | 0,21 | >0.99 | 0,21 |
| ^1^Mean (SD) or Frequency (%) |  |  |  |  |  |  |  |
| ^2^Pearson's Chi-squared test |  |  |  |  |  |  |  |
