# Supplementary material for: Biannual and Quarterly Comparison Analysis of Agglutinating Antibody Kinetics on a Subcohort of Individuals Exposed to Leptospira interrogans in Salvador, Brazil
Source: Front Med (Lausanne). 2022 Apr 14;9:862378. doi: 10.3389/fmed.2022.862378 (PMC9048256; doi:10.3389/fmed.2022.862378)
Supplement: Supplementary Table S3 — Characteristic of the subcohort group comparing the stratified analysis of infection based on the biannual analysis follow-up. [file Table_3.DOCX]

Supplementary Table 3. Characteristic of the subcohort group comparing the stratified analysis of infection based on the biannual analysis follow-up.

| **Characteristic** | **Total (N = 72)^1^** | **No infection (N = 35)^1^** | **Infection (N = 25)^1^** | **Reinfection (N = 12)^1^** | **p-value^2^  infection vs no infection** | **p-value^2^  reinfection vs no infection** | **p-value^2^  infection vs reinfection** |
| --- | --- | --- | --- | --- | --- | --- | --- |
| **Median Age (years)** | 28 (16) | 26 (20) | 30 (12) | 30 (15) | 0,3 | 0,44 | 0,99 |
| **Age (years)** |  |  |  |  | **<0.001** | **0,027** | 0,38 |
| 05-14 | 21 (29) | 16 (45.7%) | 2 (8.0%) | 3 (25.0%) |  |  |  |
| 15-24 | 9 (12) | 4 (11.4%) | 5 (20.0%) | 0 (0.0%) |  |  |  |
| 25-34 | 18 (25) | 4 (11.4%) | 9 (36.0%) | 5 (41.7%) |  |  |  |
| 35-44 | 12 (17) | 2 (5.7%) | 7 (28.0%) | 3 (25.0%) |  |  |  |
| > 44 | 12 (17) | 9 (25.7%) | 2 (8.0%) | 1 (8.3%) |  |  |  |
| **Sex** |  |  |  |  | >0.99 | >0.99 | >0.99 |
| Female | 36 (50) | 17 (48.6%) | 13 (52.0%) | 6 (50.0%) |  |  |  |
| Male | 36 (50) | 18 (51.4%) | 12 (48.0%) | 6 (50.0%) |  |  |  |
| **Ethnicity** |  |  |  |  | 0,38 | 0,39 | 0,41 |
| Black | 29 (40) | 13 (37.1%) | 9 (36.0%) | 7 (58.3%) |  |  |  |
| Brown | 33 (46) | 15 (42.9%) | 14 (56.0%) | 4 (33.3%) |  |  |  |
| White | 10 (14) | 7 (20.0%) | 2 (8.0%) | 1 (8.3%) |  |  |  |
| Others | 0 (0) | 0 (0.0%) | 0 (0.0%) | 0 (0.0%) |  |  |  |
| **Education** |  |  |  |  | >0.99 | 0,58 | 0,63 |
| Up to 9th year | 56 (78) | 28 (80.0%) | 20 (80.0%) | 8 (66.7%) |  |  |  |
| More than 9th year | 16 (22) | 7 (20.0%) | 5 (20.0%) | 4 (33.3%) |  |  |  |
| **Married or stable union** | 22 (31) | 6 (17.1%) | 11 (44.0%) | 5 (41.7%) | **0,047** | 0,18 | >0.99 |
| **Informal employment** | 36 (50) | 14 (40.0%) | 15 (60.0%) | 7 (58.3%) | 0,21 | 0,44 | >0.99 |
| **Per capita household income (US$/day)** | 4.3 (3.7) | 4.2 (4.1) | 4.4 (3.0) | 4.8 (3.8) | 0,8 | 0,6 | 0,72 |
| **Cleaned sewage** | 10 (14) | 0 (0.0%) | 6 (24.0%) | 4 (33.3%) | **0,009** | **0,003** | 0,84 |
| **Open sewage at <10m from home** | 50 (69) | 21 (60.0%) | 21 (84.0%) | 8 (66.7%) | 0,086 | 0,95 | 0,44 |
| **Accumulated trash within <10m of home** | 24 (33) | 11 (31.4%) | 11 (44.0%) | 2 (16.7%) | 0,47 | 0,54 | 0,21 |
| **Sewage contact** | 33 (46) | 15 (42.9%) | 11 (44.0%) | 7 (58.3%) | >0.99 | 0,55 | 0,64 |
| **Floodwater near home** | 40 (56) | 21 (60.0%) | 12 (48.0%) | 7 (58.3%) | 0,51 | >0.99 | 0,81 |
| **Mud near home** | 45 (62) | 20 (57.1%) | 15 (60.0%) | 10 (83.3%) | >0.99 | 0,2 | 0,3 |
| **Work in construction** | 5 (6.9) | 2 (5.7%) | 2 (8.0%) | 1 (8.3%) | >0.99 | >0.99 | >0.99 |
| **Work related to hawker** | 3 (4.2) | 1 (2.9%) | 2 (8.0%) | 0 (0.0%) | 0,76 | >0.99 | 0,82 |
| **Work related to garbage removal** | 7 (9.7) | 1 (2.9%) | 5 (20.0%) | 1 (8.3%) | 0,081 | >0.99 | 0,67 |
| **Work involves contact with mud** | 3 (4.2) | 1 (2.9%) | 2 (8.0%) | 0 (0.0%) | 0,76 | >0.99 | 0,82 |
| **Work involves contact with flood water** | 3 (4.2) | 1 (2.9%) | 2 (8.0%) | 0 (0.0%) | 0,76 | >0.99 | 0,82 |
| **Work involves sewage contact** | 3 (4.2) | 1 (2.9%) | 2 (8.0%) | 0 (0.0%) | 0,76 | >0.99 | 0,82 |
| **Fever** | 16 (22) | 5 (14.3%) | 7 (28.0%) | 4 (33.3%) | 0,33 | 0,31 | >0.99 |
| ^1^Mean (SD) or Frequency (%) | | |  | |  |  |  |
| ^2^Welch Two Sample t-test; Pearson's Chi-squared test | | |  | |  |  |  |
